# Innovative Detection of Biomarkers Based on Chemiluminescent Nanoparticles and a Lensless Optical Sensor

**DOI:** 10.3390/bios14040184

**Published:** 2024-04-09

**Authors:** Cristina Potrich, Gianluca Palmara, Francesca Frascella, Lucio Pancheri, Lorenzo Lunelli

**Affiliations:** 1 Fondazione Bruno Kessler, Center for Sensors & Devices, Via Sommarive 18, I-38123 Trento, Italy; palmara@uji.es (G.P.); lunelli@fbk.eu (L.L.); 2 Consiglio Nazionale delle Ricerche, Istituto di Biofisica, Via alla Cascata 56/C, I-38123 Trento, Italy; 3 Department of Applied Science and Technology (DISAT), Politecnico di Torino, Corso Duca degli Abruzzi 24, I-10129 Torino, Italy; francesca.frascella@polito.it; 4 Department of Industrial Engineering, University of Trento, Via Sommarive 9, I-38123 Trento, Italy; lucio.pancheri@unitn.it

**Keywords:** chemiluminescent detection, lensless biosensor, lateral flow test, antibody-functionalized nanoparticles

## Abstract

The identification and quantification of biomarkers with innovative technologies is an urgent need for the precise diagnosis and follow up of human diseases. Body fluids offer a variety of informative biomarkers, which are traditionally measured with time-consuming and expensive methods. In this context, lateral flow tests (LFTs) represent a rapid and low-cost technology with a sensitivity that is potentially improvable by chemiluminescence biosensing. Here, an LFT based on gold nanoparticles functionalized with antibodies labeled with the enzyme horseradish peroxidase is combined with a lensless biosensor. This biosensor comprises four Silicon Photomultipliers (SiPM) coupled in close proximity to the LFT strip. Microfluidics for liquid handling complete the system. The development and the setup of the biosensor is carefully described and characterized. C-reactive protein was selected as a proof-of-concept biomarker to define the limit of detection, which resulted in about 0.8 pM when gold nanoparticles were used. The rapid readout (less than 5 min) and the absence of sample preparation make this biosensor promising for the direct and fast detection of human biomarkers.

## 1. Introduction

The advent of precision medicine in recent years has brought to light the huge potentialities but also the challenges of this approach for disease prevention, diagnosis, and treatments customized to the individual patient [1,2]. Liquid biopsy in particular is recognized as a powerful tool for this approach, consisting in a minimally invasive blood test aimed at measuring the individual molecular profile of patients over time [3,4]. Being minimally invasive, this test can be indeed serially repeated, giving information about the pathological and physiological state of an individual in real time. Diverse clinical sources of biomarkers such as urine [5] and saliva [6] and especially blood, mainly for biomarkers related to cancer [3], are described in the literature. Several techniques have been proposed for cancer molecular profiling with a liquid biopsy approach [7], but new, more versatile technologies are needed to move a step forward and bring the liquid biopsy in the clinical practice. Highly sensitive, rapid, easy-to-use tests would be essential in this context and biosensors could offer a valuable option for the precise identification of biomarkers.

Recent years have witnessed incredible advances in biosensor design and development [8]. Biosensors made of various materials [9,10], hosting different detection methodologies [11,12,13], and devoted to different applications, from healthcare [13,14] to food safety [15], have been proposed. Among the different detection methods, chemiluminesence-based detection has recently gained great attention for the possibility to achieve ultrasensitive detection [16]. Chemiluminescence have also been suggested as a smart detection method for lateral flow tests (LFT) measuring salivary [17] or serum biomarkers [18,19].

LFTs are paper-based devices which take advantage of a chromatographic system, which allows the migration of a biological sample *via* capillary forces [20,21]. The different molecules present in the sample move across the reaction membrane passing through at least two lines where specific binding elements are deposited. The interaction of the molecule to be detected (biomarker) with its specific binding element occurs on the first line encountered during the migration (i.e., test line, TL), while the interaction of control molecules with the last line of the test indicated that the test has been correctly executed (control line, CL). The specific recognition of the biomarker occurs mainly by an immunochemical reaction, i.e., between an antibody–antigen interaction. LFTs have been widespread employed for decades, since they respond to all the “ASSURED” criteria (affordable, sensitive, specific, user-friendly, rapid/robust, equipment-free, and deliverable to end-users), as defined by the World Health Organization [16,18].

For many applications, colorimetric LFTs are sufficiently effective to cover the required dynamic range, or alternatively, their advantages in terms of low cost, rapidity and usability outpace the limits of sensitivity. However, some applications require an improved sensitivity and therefore the overcoming of the standard detection methods is desirable. In fact, the readings of colorimetric LFTs being visible even by eye and thus being directly observable [22] is quite common, but this usually leads to qualitative results. Over the years, different methodologies have been used in order to improve LFTs’ sensitivity. For example, fluorescence- and chemical-luminescence-based approaches have proved to be useful in enhancing the detection limit of these devices [20,23] and remarkable results were also obtained with the inclusion of nanomaterials such as nanoparticles, carbon nanotubes, quantum dots, or other nanomaterials [20]. Here, an LFT based on gold nanoparticles as an enhancing system and on the chemiluminescence detection of C-reactive protein (CRP), selected as proof-of-concept protein biomarker, has been developed. CRP is a homopentameric acute-phase inflammatory protein highly expressed during inflammatory conditions [24,25] and is therefore widely tested as a diagnostic biomarker and for the monitoring of inflammatory conditions [26].

The chemiluminescence signal exploited in this work is generated by the luminescent transition from excited-state molecules, whose excitation is catalyzed by the enzyme horseradish peroxidase (HRP) in the presence of opportune reagents [27]. An HRP enzyme conjugated to an antibody able to recognize the CRP protein (a-CRP-HRP) was employed. Two different ways of immobilizing the a-CRP-HRP complex on the LFT conjugate pad are compared: (a) direct immobilization of the complex, and (b) immobilization of a-CRP-HRP molecules on gold nanoparticles, which are in turn immobilized on the conjugate pad. The developed chemiluminescence signal is detected with an array of four silicon photomultipliers (SiPM), positioned in close proximity to the LFT lines. SiPMs are solid state photodetectors capable of sensing, timing, and quantifying low-light signals down to the single-photon level. They are made of arrays of integrated single-photon avalanche diodes (SPADs) [28], whose suitability for chemiluminescence-based detection of circulating biomarkers has already been demonstrated [29]. The use of a high-sensitivity photodetector operating at room temperature with an area of the order of square millimeters, such as a SiPM, allows to remove any lens or optical system or cooling need with the huge advantage of obtaining a low-cost, simple, compact, and ultrasensitive device [30,31,32,33].

## 2. Materials and Methods

### 2.1. Materials

Glass fiber sample pad strips (Millipore GFCP103000, Burlington, MA, USA), glass fiber conjugate pad strips (Millipore GFDX083000), Whatman^®^ FF170HP sheets nitrocellulose membrane, cellulose fiber absorbent pad sheets (Millipore CFSP223000), C-reactive protein from human fluids (CRP), and peroxidase from horseradish (HRP) were purchased from Merk Life Science S.r.l. (Milan, Italy). Anti-CRP polyclonal goat IgG antibody Horse Radish Peroxidase conjugated (ThermoFisher Scientific, Waltham, MA, USA), a-CRP-HRP in the following, was used for conjugation to 40 nm gold nanoprticles (gNPs; Gold Conjugation Kit (40 nm, 20 OD); abcam, Cambridge, UK). Anti-CRP mouse monoclonal antibody (abcam) and recombinant Protein G (ThermoFisher Scientific) were spotted on nitrocellulose as test line (TL) and control line (CL), respectively. The developer solutions were: SuperSignal™ELISA Femto Substrate from ThermoFisher Scientific, Westar SuperNova from Cyanagen S.R.L. Bologna, Italy and Westar HyperNova also from Cyanagen S.R.L. Bologna, Italy.

### 2.2. Setup of the Biosensor

**Figure 1:**
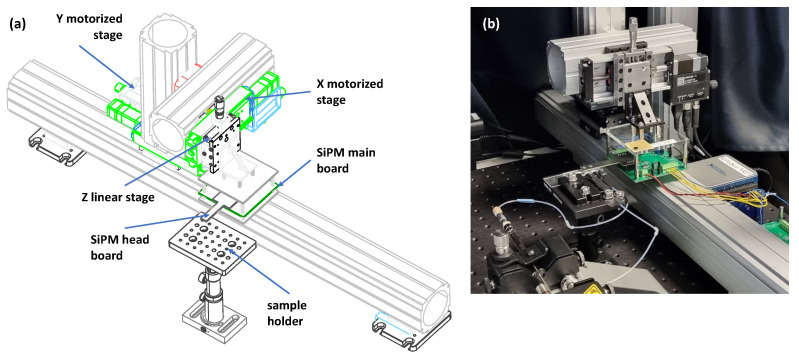
Mechanical layout of the device. (**a**) Sketch of the biosensors: movement mechanism, sample positioning and board. (**b**) Picture of the complete device.

**Figure 2:**
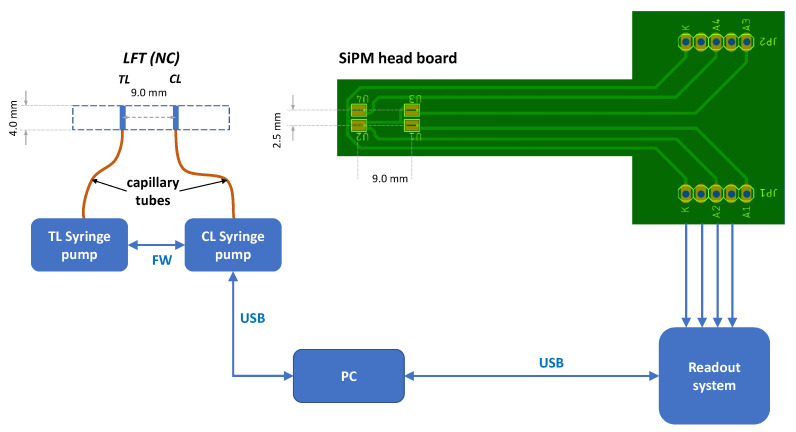
Layout of the connections of the biosensor, showing the main block components. (**Left**) developer delivery subsystem, (**Right**) the SiPM head board connections.

The device described in this study comprises a lateral flow test built in house, a detector based on four Silicon Photomultipliers (SiPMs; model ASD-NUV1S-P, AdvanSiD, Trento, Italy), hardware and software to adjust the sensor head position so that it can be positioned in close proximity to the LFT for an optimal reading, and microfluidics for the handling of the developer solution. Rails and accessories were from Thorlabs XT66 series (Thorlabs Inc., Newton, NJ, USA), while motorized stages were models X-LHM050A (*X*-axis) and X-LSM050A (*Y*-axis) from Zaber (Zaber, Vancouver, BC, Canada). A sketch of the device is illustrated in Figure 1a, where the XYZ mechanical movements are highlighted together with the sample holder (LFT strip holder) and the SiPM main board. The four SiPMs mounted on a secondary board (head board) are shown in Figure 2, which reports also the distances among the SiPMs, optimized for measuring at the same time both the TL and the CL, given the geometry of the LFT. A picture of the device is shown in Figure 1b, where connections (microfluidic ChipShop, Jena, Germany) and tubes (0.25 mm internal diameter; IDEX Health & Science LLC, Saitama, Japan) are also visible. Tubes connect the LFT holder to two microsyringe pumps (Legato 185, KD Scientific, Holliston, MA, USA) used to inject simultaneously the chemiluminescence developing solution to the two lines of the LFT. The developing solution is inserted in two 500 µL Hamilton 1700 Gas Tight syringes, actuated by the microsyringe pumps. All the described equipment was kept in the complete dark during measurements.

The layout of the electrical connections of the four SiPMs to the head board and to the readout system and the PC as well as a schematic drawing of the developer delivery system and its connections is explained in Figure 2. Further details of the readout system box are described in the following Section 3.1 and in the following figures. Both the syringe pumps and the SiPMs are controlled by programs running on a laptop PC. Syringe pumps are controlled via a a Python script that sends control codes through a USB port to the main syringe pump, while the second syringe pump is connected to the first one with a FireWire cable. A header of each command is used to direct the command to the intended syringe pump. When the system is initialized, the SiPM reading program (written in LabView) reads the dark currents of the SiPM that will be then subtracted from data.

### 2.3. Preparation of Gold Nanoparticles

The chemiluminesce signal exploited in this work for the detection of the CRP biomarker is generated by the enzyme HRP conjugated to the specific antibody a-CRP-HRP. In the presence of suitable substrates, HRP catalyzes a reaction that generates an excited-state product that emits light in the blue region when decays to its ground state (Figure S1A). The a-CRP-HRP antibody is bound to the nanoparticles (with the protocol described below) and recognizes the CRP migrated on the LFT. When the chemiluminescence substrates are added to the LFT, a chemiluminescence signal is generated.

The specific antibody a-CRP-HRP was conjugated to the gold nanoparticles, following manufacturer’s instructions. Briefly, 1 µg of antibody was added to the reaction buffer given in the conjugation kit and the mixture was transferred to a vial provided by the kit, containing the gNPs. After 15 min at room temperature, a quencher reagent provided by the kit was added for 5 min to stop the reaction. Next, the unbound antibody was removed by washing the nanoparticles with PBS added with 1/10 *v*/*v* of quencher and centrifuging them at 9000× *g* for 10 min. Finally, the pellet was suspended in one volume of PBS added with the diluted quencher.

Moreover, the amount of a-CRP-HRP bound to gNPs that is effective in generating the chemiluminescence signal was estimated as follows. Firstly, the chemiluminescence signal of known amounts of free a-CRP-HRP in solution were determined and used for the construction of a calibration curve. Then, the chemiluminescence of free a-CRP-HRP still present in the washing solution, after the coupling reaction described above, was measured and the amount of a-CRP-HRP was inferred from the calibration curve. This value allows the estimation of the amount of a-CRP-HRP that is bound to gold nanoparticles ($Ab_{bound}$), by subtracting the a-CRP-HRP still present in the washing solution from the total a-CRP-HRP initially added to the reaction vial. Finally, by measuring the chemiluminescence of a known amount of gold nanoparticles conjugated with a-CRP-HRP, and using the calibration curve previously measured, one can estimate an effective value of bound a-CRP-HRP ($Ab_{effective}$). This value may differ from the value $Ab_{bound}$ due to a different capability to catalyze the chemiluminescence reaction of the bound a-CRP-HRP, with respect to the free a-CRP-HRP.

The chemiluminescence developed during this test was measured with the ChemiDoc™ Imaging System (BioRad), adding the developer solution to the samples, while the obtained data were elaborated with the Fiji software (version 2.1.0, created by Johannes Schindelin, Albert Cardona and Pavel Tomancak) [34].

Thee different kits for the development of the chemiluminescence reaction were tested. The chemiluminescence spectra were acquired for the three substrates in the presence of 16.5 ng/mL of HRP and are compared in Figure S1B. Spectra were acquired with the SPEX FluorMax spectrofluorimeter (Horiba Instruments Inc., Edison, NJ, USA) in emission mode and without lamp excitation. Chemiluminescence spectra were recorded from from 320 to 700 nm, before and after the addition of the HRP enzyme.

### 2.4. Fabrication of the LFT Strips

Components of the LFT are cut with a guillotine paper cutter and manually assembled, using an adhesive plastic back for sample pad, conjugate pad and absorbent pad. Nitrocellulose strips (cut from A4 sheets) already have their own plastic back. All parts (and the assembled LFTs) have a width of 4 mm.

The first step for the fabrication of LFT consists in cutting the conjugate pad strips to the length of 4 mm and the sample pad strips in 2 cm long pieces. These two types of pads are then treated with a solution of 5% sucrose and 0.5% Tween^®^ 20 for the conjugate pad and with a solution of 1% bovine serum albumin (BSA) and 0.5% Tween^®^ 20 for the sample pad, to passivate them and also allow a passivation of the nitrocellulose during the sample migration. This passivation treatment was setup and optimized empirically, starting from standard conditions reported in the literature [22]. The two pads are incubated for 2 h at 37 °C to allow a suitable stabilization of reagents on the pad material.

**Figure 3:**
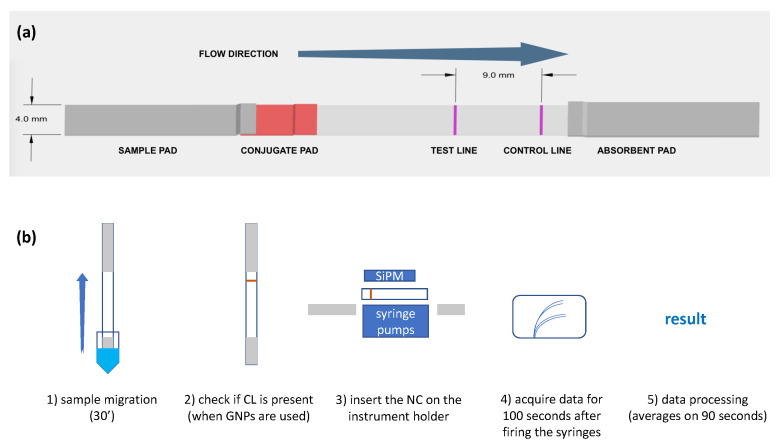
Scheme of assembled LFT, evidencing the different areas and dimensions of the strip (**a**) and sketch of the experiment (**b**).

The conjugate pad was loaded with 1 µL of functional gNPs (conjugated with the antibody see Section 2.3), diluted 30 times in PBS and let dry at 37 °C for two hours. For experiments without gNPs, 0.72 nM of a-CRP-HRP, was added to the conjugate pad, i.e., a quantity that gives rise to the same chemiluminescence signal of a solution containing 1 µL of functional gNPs. A test line and control line were then spotted on the nitrocellulose strips with the Biodyssey Calligrapher Spotter (Biorad, MCP310S solid pins), starting from 2 mg/mL concentration of anti-CRP antibody and 0.5 mg/mL protein G, respectively. The two lines were spotted at the same time, mounting on the spotter head two pins, leaving an empty position in-between, which corresponded to a distance of 9 mm between lines. The spotting protocol was adjusted in order to obtain spotted lines of 4 mm in length and around 0.7 mm in width (see Supplementary Figures S2 and S3). Next, the LFT strips were assembled as depicted in Figure 3a. The passivated sample pad and the conjugate pad containing either gNPs or a-CRP-HRP were slightly overlapped so that the conjugate pad was also overlapped to the nitrocellulose strip. The latter was 3 cm long and exposes the TL and CL, which are 9 mm apart. The LFT strip was completed by a 2 cm long absorbent pad strip. LFT strips were prepared and used the same day of the experiment.

### 2.5. Detection Protocol of the CRP Biomarker

The LFT strips fabricated as described in the previous paragraph were used for the detection of the CRP biomarker. The protocol, schematized in Figure 3b, involves the insertion of the strip in a 0.2 mL vial containing the solution of CRP at different concentrations (step 1 in Figure 3b). The solution was allowed to migrate for 30 min; during this time, a band corresponding to the CL becomes visible if gNPs were used in the experiment (step 2 in Figure 3b). At the end of migration, the portion of strip containing the bands to measure, i.e., the nitrocellulose part, was inserted in the sample holder of the device shown in Figure 1 (step 3 in Figure 3b) and the signal without developer (background) was acquired for 90 s. Then, 5 µL of the chemiluminescence developing substrates were delivered simultaneously on both lines in the dark and the chemiluminescence signal developed on the two lines was acquired for at least 100 s in real time by the four SiPMs (step 4 in Figure 3b). Finally, data are analyzed (step 5 in Figure 3b), averaging the signal recorded for 90 s.

## 3. Results and Discussion

### 3.1. Description of the SiPM-Based Detector

**Figure 4:**
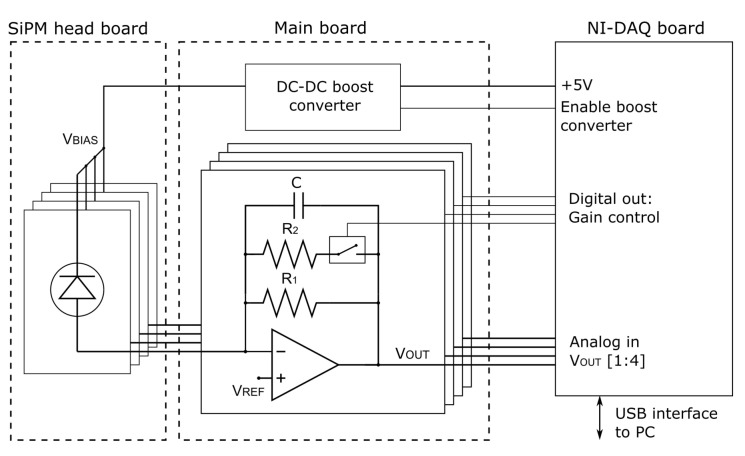
Readout system schematic diagram, see text for details.

A simplified schematic diagram of the readout system is shown in Figure 4. The system is made of the SiPM head board, a main board including amplifiers and ancillary electronics, and a USB-6002 National Instrument Data-Acquisition Board (DAQ) interfaced with the main board and with a PC, generating the required control signals and handling data logging operations. The choice of SiPMs was dictated by the need to have detectors with a relatively large area to maximize the numerical aperture of the lensless readout system working in proximity, while maintaining very low noise. The high gain of the SiPMs makes the electronics readout noise negligible if compared to their internal noise, essentially determined by Dark Count Rate and correlated noise components. Since the application did not require a large bandwidth readout, a transimpedance scheme with low-pass filtering characteristics was chosen. Four AdvanSiD NUV-SiPMs (model ASD-NUV1S-P) were mounted on the head board. The devices feature an active area of 1 mm^2^, a Photon Detection Efficiency > 40% at 425 nm and a Dark Count Rate < 100 kHz/mm^2^. The signal of each of the four SiPMs was amplified by a transimpedance amplifier with programmable gain. The high-voltage bias required for SiPM operation was generated by a DC-DC boost converter starting from the 5 V supply provided by the DAQ board. Digital control signals generated by the DAQ board were used to define the amplifier gain and to enable/disable the boost converter. Custom software was created to handle board control and data acquisition operations. The amplifiers’ output signals were acquired at a sample rate of 1 kS/s. The samples acquired within each second were then averaged to improve the Signal-to-Noise ratio, providing a final data stream with 1 datapoint/s for each sensor.

### 3.2. Setup and Characterization of the LFT

**Figure 5:**
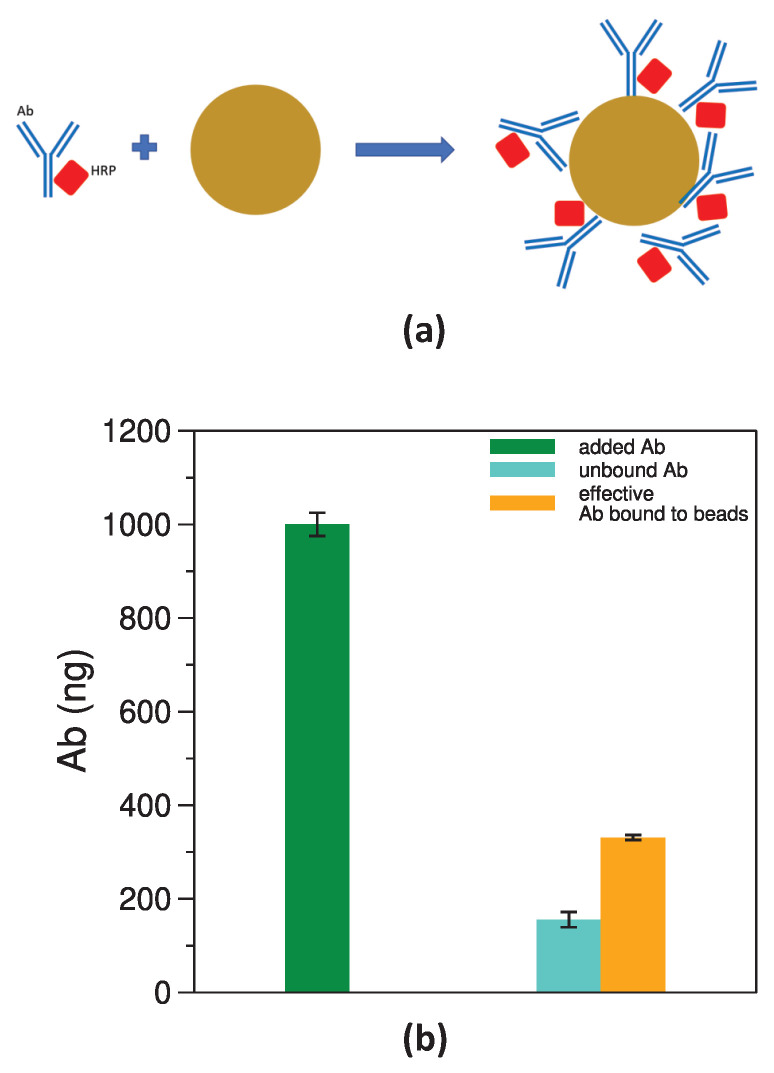
Scheme of the gNPs’ functionalization with a-CRP-HRP (**a**) and comparison of the chemiluminescence activity of the a-CRP-HRP in solution and bound to gNPs (**b**). The green bar represents the amount of Ab added to the coupling reaction, while the pale green bar corresponds to the amount of unbound Ab. The gold bar refers to a-CRP-HRP bound to gNPs and effective in generating chemiluminescence, not to be confused with a-CRP-HRP simply bound to gNPs calculated from the difference between the green and pale green bars. See Section 2.3 for calculation details.

LFT strips were assembled optimizing the different steps from passivation to the amount of spotted antibody and protein G to the type and volume of solutions for developing the chemiluminescence signal. The developed LFT is based on gNPs functionalized with anti-CRP antibody conjugated with HRP with a reaction is summarized in Figure 5a. CRP is a protein biomarker highly informative about inflammatory conditions and one of the most requested tests in laboratory medicine [26]. For this reason, CRP was selected as proof-of-concept biomarker in this study. The anti-CRP antibody was made to react with the gold nanoparticles, obtaining structures that expose the antibody molecules with different orientations (Figure 5a).

The functionalization of gNPs was checked in terms of chemiluminescence-effective antibody molecules present on the surface of the particles. The washing solution deriving from the conjugation of antibody to gNPs as described in Section 2.3 and the functionalized gNPs diluted to suitable amounts were reacted with the developing solution, in parallel with known amounts of antibody HRP conjugated. The HRP enzyme present on the conjugated antibodies processes the chemiluminescence substrates, producing a signal that is measured for all samples and compared to the signal emitted by known amounts of the same antibody measured in the same conditions. This computation allowed us to estimate the amount of antibody present in the volume added to gNPs at the beginning of the functionalization protocol (“added Ab” in Figure 5b, green bar) and the unreacted antibody remaining in the washing fraction (“unbound Ab” in the same figure, pale green bar) and also to estimate the amount of antibody effective in processing the chemiluminescence substrate and which is bound on the surface of the functionalized gNPs (“effective Ab bound to beads” in Figure 5b, gold bar). Around 17% of the added antibody is unbound and remains in the washing solution. Therefore, around 83% of the added antibody is expected to be bound to the gNPs, but only 29% is directly measured by the chemiluminescence reaction, as clearly visible in Figure 5b, gold bar. It should be noted that the quantification obtained with this method is not absolute, since it measure the amount of HRP conjugated to the antibody that is effectively able to process the substrate. The antibody bound to gNPs indeed assumes different orientations, as illustrated in Figure 5a, and not all orientations expose the HRP enzyme with the same efficacy in performing the chemiluminescence reaction. As a result, a reduced amount of antibody on gNPs is quantified from the chemiluminescence signal (29% in place of 83%), meaning that only (on average) 35% of the a-CRP-HRP molecules present on gNPs is functional and able to develop an effective chemiluminescence signal.

Furthermore, the interaction of gNPs with two key components of the LFT was assessed with AFM. The AFM images (see Figure S4) show that gNPs are distributed quite homogeneously along the conjugate pad fibers. Moreover, nanoparticles dimensions are found to be in good agreement with their nominal size (40 nm) (Figures S4 and S5).

### 3.3. Implementation of the LFT for Biomarkers Detection

**Figure 6:**
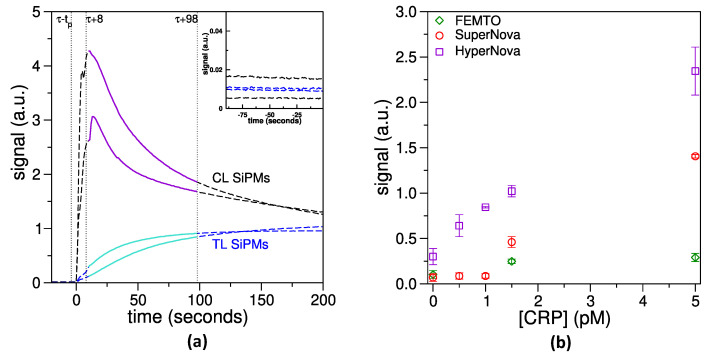
LFT implementation. (**a**): Typical response curves measured on LFT after developer injection, obtained with 0.5 pM CRP migrated along the strip. The time when signals start to rise is set to 0 (around 2–3 s after the start of pumps), while the 90 s time interval (full curves, from 8 to 98 s) represents the data that were used for processing the results. Inset: background signal, acquired at the beginning of each experiment, before delivering the developer. The vertical scale is enlarged 100 times. (**b**): Comparison of the effect of Femto, SuperNova and HyperNova chemiluminescence substrates on the signal of LFT with functional gNPs.

Once the different components of the LFT and the reading device were characterized and optimized, a complete experiment was performed. CRP diluted in buffer at known concentrations was allowed to migrate along the LFT strip for 30 min and then the NC was positioned on the device holder for measuring with the SiPM detector. Two SiPMs acquire the signal coming from the test line and two the signal from the control line. The signal was firstly measured in the dark for about 90 s (Figure 6a, inset) to set the background conditions and check the experimental setup. The background was then subtracted, but, as clearly visible in the inset of Figure 6a, its contribution is negligible in comparison with the obtained signals. Actually, the standard deviation of the background signal is around $5\times 10^{-4}$, i.e., nearly four order of magnitude lower than the detected signals. Next, 5 µL of the developing solution were injected on TL and CL simultaneously through two programmable syringe pumps and the increase in the chemiluminescence signal was followed in real time. An example of the resulting graph is reported in Figure 6a.

The development of chemiluminescence was due to the reaction promoted by the HRP enzyme conjugated to the anti-CRP antibody, which was present on the gNPs surface. This antibody is captured by protein G in the case of the CL (positive control) and by CRP, in turn immobilized by the antobodies previously spotted on the line (see Section 2.4), in the case of the TL. Protein G is indeed well known for its strong binding properties towards immunoglobulins [35], and therefore is widely used in tests based on antibodies. For this reason, protein G was selected for the CL in setting up this LFT. The TL, instead, is formed by a monoclonal anti-CRP antibody which recognizes a different epitope of CRP with respect to the polyclonal a-CRP-HRP conjugated to the gNPs. In this way, when CRP is present in the sample flowing through the LFT strip, a sandwich of CRP between the two antibodies is formed and a chemiluminescence signal is developed also at the TL position.

The signal related to CL increased very quickly (Figure 6a, purple curves) due to the high number of HRP present on this line, but also decreased quickly, since the HRP enzyme rapidly consumes the substrate present in the developing solution. On the contrary, the signal related to TL increased more slowly (Figure 6a, cyan curves) for this concentration of CRP, but it was stable for a longer time. Since the slope of these curves changes with the concentration of the biomarker to detect, a signal with an average duration of 90 s was used to calculate the signal related to a specific concentration of CRP (see Figure 6a for a visual explanation). This method allowed the construction of signal *versus* concentration graphs, as shown in Figure 6b. The developed chemiluminescence signal was also influenced by the biochemical protocol used, in particular by the developing solutions selected to trigger the chemiluminescence. Three different kits were compared in same conditions (Figure 6b), observing that the kit HyperNova performed much better than the other kits tested, in all the range of CRP concentrations explored. The HyperNova kit was therefore selected for developing all the next LFTs.

### 3.4. Estimation of the Limit of Detection

**Figure 7:**
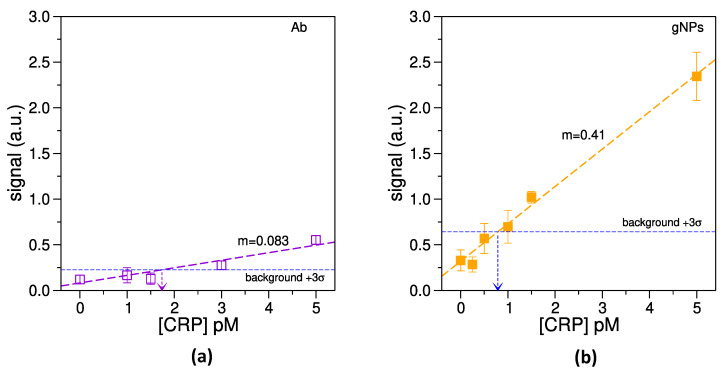
Estimation of LOD. (**a**): LOD measured for LFT performed with pure a-CRP-HRP; (**b**): LOD measured for LFT with the functionalized gNPs.

A set of measurements with the optimized reading setup and biochemical conditions was performed in order to assess the limit of detection of the device. Two different kinds of LFT were prepared: (i) with conjugate pads endowed with functionalized gNPs, and (ii) with conjugate pads treated with an equivalent amount of a-CRP-HRP, as calculated from the data reported in Section 3.2, to obtain the same capability of developing chemiluminescence in both cases. Different concentrations of CRP were then migrated on the two kinds of LFT. When functional gNPs were used as reporting material (Figure 7b), the developed signal increased substantially with respect to the use of pure antibody (Figure 7a). In other words, the presence of gNPs significantly increased the sensitivity of LFTs, as clearly visible in Figure 7. However, a slight increase in the measured signal (and of its standard deviation) was also observed even in the absence of CRP (i.e., “0” concentration in Figure 7b). Therefore, in this condition the LOD improves a bit less than expected from the increase in the slope of the fit reported in Figure 7b. The standard deviation of the signal when CRP concentration is 0 enters indeed in the LOD computation, as shown in Equation (1), where $StDev_{0}$ is the standard deviation of the signal, when no CRP is present in the sample.

(1) $$LOD=\frac{3\cdot StDev_{0}}{m}$$

As a result, the LOD obtained for LFTs employing a-CRP-HRP was around 1.8 pM (0.21 ng/mL), while it was found to be 0.8 pM ($9.4\times 10^{-2}$ ng/mL) when the same amount of effective antibody was bound to gNPs. Therefore, the use of functionalized gNPs produced an enhancement of about 2.3 times the limit of detection, and an increased chemiluminescence signal of almost five times, which could be crucial when low amounts of biomarkers have to be detected with a simple biosensor. Noticeably, comparing the error bars of the obtained signal (Figure 7) with the noise introduced by the SiPM (see Section 3.3), the LOD limiting factor results in the residual aspecific adhesion of the functionalized nanoparticles that occurs also at [CRP] = 0.

Moreover, the percentage relative standard deviation (RSD) was calculated for both conditions shown in Figure 7. When a-CRP-HRP was absorbed on the conjugate pad, RSD ranged from 50 to around 10%, while when gNPs conjugated to a-CRP-HRP were adsorbed, this parameter ranged from 35 to around 10%. In both cases a linear decreasing trend of RSD versus CRP concentration was observed.

Concerning CRP, since a concentration between 3 and 10 mg/L (i.e., about 25–83 nM) indicates only a low-grade inflammation [36], the above reported sensitivity is not really needed. However, the early detection of low amounts of other kinds of protein biomarkers, such as for example cancer biomarkers, is crucial for a positive outcome of these pathologies. CRP could be indeed considered as a suitable proof-of-concept biomarker to facilitate the setup of a sensitive, rapid, easy-to-use test.

It may also be worth noting that the use of functional gNPs allows the detection of the correct formation of the control line by eye after sample migration before developing the chemiluminescence signal. Using pure antibody always requires instead a complete measurement, even to only appreciate the presence of the CL, increasing the time and costs of the test. Moreover, if the concentration of the biomarker greatly exceeds the LOD, the TL becomes visible by eye (see Supplementary Figure S6), joining the advantages of traditional LFTs in terms of low costs and straightforward detection with the high sensitivity of chemiluminescence. The LFT presented in this paper was also compared to other methods for the detection of CRP biomarker (Table S1), finding that the LOD is improved by around one order of magnitude with respect to traditional LFT and capillary ELISA [37,38]. More sensitive detection methods do exist [39], but are more complicated in comparison to the method presented here.

## 4. Conclusions

The concept of liquid biopsy offers huge possibilities for a non-invasive diagnosis of several diseases and for the follow up of disease treatments, but poses also several challenges. The need of traditional laboratories and skilled personnel to perform liquid biopsy tests is one of the most critical. Here, a simple assay based on LFT and SiPM detectors is proposed with the final goal being to fabricate a highly sensitive LFT based on functionalized gNPs and a reading system based on SiPMs. The presence of functionalized gNPs led to a five-fold increase in the measured chemiluminescence signal, while the use of SiPMs showed the good potentialities of a compact system not requiring lenses or cooling for measuring the signal, even when detecting low-concentrated biomarkers, allowing the minimization of the detector’s dimensions. This aspect is crucial for preparing point-of-care tests that are really usable in clinical settings, when low concentrations of circulating biomarkers have to be detected.

## Data Availability

The data presented in this study are available from the corresponding author upon request.

## Supplementary Materials

The following are available online at https://www.mdpi.com/article/10.3390/bios14040184/s1, Figure S1: Chemiluminescence catalytic reaction and luminescence spectra of the three developer solutions, Figure S2: Adjustment of spotting parameters, Figure S3: Characterization of spotted lines, Figure S4: AFM data of the gNPs on the conjugate pad, Figure S5: Characterization by AFM of the nitrocellullose membrane, previously spotted with protein G and passivated, Figure S6: Picture showing CL and TL visible by eye. Table S1: Comparison of the performances of some CRP detection systems [37,38,39,40,41].

## References

[1] Hasanzad M., Sarhangi N., Chimeh S.E., Ayati N., Afzali M., Khatami F., Nikfar S., Meybodi H.R.A. (2022). Precision medicine journey through omics approach. J. Diabetes Metab. Disord..

[2] Liu X., Luo X., Jiang C., Zhao H. (2019). Difficulties and challenges in the development of precision medicine. Clin. Genet..

[3] Lianidou E., Pantel K. (2019). Liquid biopsies. Genes Chromosom. Cancer.

[4] Battistelli M. (2021). Liquid Biopsy: A Family of Possible Diagnostic Tools. Diagnostics.

[5] Oshi M., Murthy V., Takahashi H., Huyser M., Okano M., Tokumaru Y., Rashid O.M., Matsuyama R., Endo I., Takabe K. (2021). Urine as a Source of Liquid Biopsy for Cancer. Cancers.

[6] Aro K., Wei F., Wong D.T., Tu M. (2017). Saliva Liquid Biopsy for Point-of-Care Applications. Front. Public Health.

[7] Nikanjam M., Kato S., Kurzrock R. (2022). Liquid biopsy: Current technology and clinical applications. J. Hematol. Oncol..

[8] Chadha U., Bhardwaj P., Agarwal R., Rawat P., Agarwal R., Gupta I., Panjwani M., Singh S., Ahuja C., Selvaraj S.K. (2022). Recent progress and growth in biosensors technology: A critical review. J. Ind. Eng. Chem..

[9] Naresh V., Lee N. (2021). A Review on Biosensors and Recent Development of Nanostructured Materials-Enabled Biosensors. Sensors.

[10] Li T., Shang D., Gao S., Wang B., Kong H., Yang G., Shu W., Xu P., Wei G. (2022). Two-Dimensional Material-Based Electrochemical Sensors/Biosensors for Food Safety and Biomolecular Detection. Biosensors.

[11] Cho I.H., Kim D.H., Park S. (2020). Electrochemical biosensors: Perspective on functional nanomaterials for on-site analysis. Biomater. Res..

[12] Altug H., Oh S.H., Maier S.A., Homola J. (2022). Advances and applications of nanophotonic biosensors. Nat. Nanotechnol..

[13] Kim J., Campbell A.S., de Ávila B.E., Wang J. (2019). Wearable biosensors for healthcare monitoring. Nat. Biotechnol..

[14] Mohankumar P., Ajayan J., Mohanraj T., Yasodharan R. (2021). Recent developments in biosensors for healthcare and biomedical applications: A review. Measurement.

[15] Lan L., Yao Y., Ping J., Ying Y. (2017). Recent advances in nanomaterial-based biosensors for antibiotics detection. Biosens. Bioelectron..

[16] Roda A., Arduini F., Mirasoli M., Zangheri M., Fabiani L., Colozza N., Marchegiani E., Simoni P., Moscone D. (2020). A challenge in biosensors: Is it better to measure a photon or an electron for ultrasensitive detection?. Biosens. Bioelectron..

[17] Zangheri M., Cevenini L., Anfossi L., Baggiani C., Simoni P., Nardo F.D., Roda A. (2015). A simple and compact smartphone accessory for quantitative chemiluminescence-based lateral flow immunoassay for salivary cortisol detection. Biosens. Bioelectron..

[18] Han G., Kim M. (2020). Highly Sensitive Chemiluminescence-Based Lateral Flow Immunoassay for Cardiac Troponin I Detection in Human Serum. Sensors.

[19] Kim H.T., Jin E., Lee M.H. (2021). Portable Chemiluminescence-Based Lateral Flow Assay Platform for the Detection of Cortisol in Human Serum. Biosensors.

[20] Bahadir E.B., Sezgintürk M.K. (2016). Lateral flow assays: Principles, designs and labels. Trends Anal. Chem..

[21] Quesada-González D., Merkoçi A. (2015). Nanoparticle-based lateral flow biosensors. Biosens. Bioelectron..

[22] Raphael Wong H.T. (2009). Lateral Flow Immunoassay.

[23] Roda A., Mirasoli M., Michelini E., Fusco M.D., Zangheri M., Cevenini L., Roda B., Simoni P. (2016). Progress in chemical luminescence-based biosensors: A critical review. Biosens. Bioelectron..

[24] Sproston N.R., Ashworth J.J. (2018). Role of C-Reactive Protein at Sites of Inflammation and Infection. Front. Immunol..

[25] Volanakis J. (2001). Human C-reactive protein: Expression, structure, and function. Mol. Immunol..

[26] Plebani M. (2023). Why C-reactive protein is one of the most requested tests in clinical laboratories?. Clin. Chem. Lab. Med..

[27] Marquette C.A., Blum L.J. (2006). Applications of the luminol chemiluminescent reaction in analytical chemistry. Anal. Bioanal. Chem..

[28] Gundacker S., Heering A. (2020). The silicon photomultiplier: Fundamentals and applications of a modern solid-state photon detector. Phys. Med. Biol..

[29] Pasquardini L., Pancheri L., Potrich C., Ferri A., Piemonte C., Lunelli L., Napione L., Comunanza V., Alvaro M., Vanzetti L. (2015). SPAD aptasensor for the detection of circulating protein biomarkers. Biosens. Bioelectron..

[30] Ghosh S., Aggarwal K., Vinitha T. U., Nguyen T., Han J., Ahn C.H. (2020). A new microchannel capillary flow assay (MCFA) platform with lyophilized chemiluminescence reagents for a smartphone-based POCT detecting malaria. Microsyst. Nanoeng..

[31] Ruffinatti F.A., Lomazzi S., Nardo L., Santoro R., Martemiyanov A., Dionisi M., Tapella L., Genazzani A.A., Lim D., Distasi C. (2020). Assessment of a Silicon-Photomultiplier-Based Platform for the Measurement of Intracellular Calcium Dynamics with Targeted Aequorin. ACS Sens..

[32] Calabretta M.M., Montali L., Lopreside A., Fragapane F., Iacoangeli F., Roda A., Bocci V., D’Elia M., Michelini E. (2021). Ultrasensitive On-Field Luminescence Detection Using a Low-Cost Silicon Photomultiplier Device. Anal. Chem..

[33] Yu Y., Nie W., Chu K., Wei X., Smith Z.J. (2023). Highly Sensitive, Portable Detection System for Multiplex Chemiluminescence Analysis. Anal. Chem..

[34] Schindelin J., Arganda-Carreras I., Frise E., Kaynig V., Longair M., Pietzsch T., Preibisch S., Rueden C., Saalfeld S., Schmid B. (2012). Fiji: An open-source platform for biological-image analysis. Nat. Methods.

[35] Akerström B., Björck L. (1986). A physicochemical study of protein G, a molecule with unique immunoglobulin G-binding properties. J. Biol. Chem..

[36] Antonelli M., Kushner I. (2017). It’s time to redefine inflammation. FASEB J. Off. Publ. Fed. Am. Soc. Exp. Biol..

[37] Kim W., Cho H.Y., Jeong B., Byun S., Huh J., Kim Y.J. (2018). Synergistic Use of Gold Nanoparticles (AuNPs) and “Capillary Enzyme-Linked Immunosorbent Assay (ELISA)” for High Sensitivity and Fast Assays. Sensors.

[38] Oh Y.K., Joung H.A., Han H.S., Suk H.J., Kim M.G. (2014). A three-line lateral flow assay strip for the measurement of C-reactive protein covering a broad physiological concentration range in human sera. Biosens. Bioelectron..

[39] Zhang J., Zhang W., Guo J., Wang J., Zhang Y. (2017). Electrochemical detection of C-reactive protein using Copper nanoparticles and hybridization chain reaction amplifying signal. Anal. Biochem..

[40] CRP Human Instant ELISA™ Kit. https://www.thermofisher.com/elisa/product/CRP-Human-Instant-ELISA-Kit/BMS288INST.

[41] Human CRP ELISA Kit (C-Reactive protein), Fluorescent. https://www.abcam.com/en-it/products/elisa-kits/human-crp-elisa-kit-c-reactive-protein-fluorescent-ab278042#tab=support.

