# Supplementary material for: Innovative Detection of Biomarkers Based on Chemiluminescent Nanoparticles and a Lensless Optical Sensor
Source: Biosensors (Basel). 2024 Apr 9;14(4):184. doi: 10.3390/bios14040184 (PMC11048690; doi:10.3390/bios14040184)
Supplement: Supplementary file 1 [file biosensors-14-00184-s001.zip › biosensors-2875673-supplementary.pdf]

Innovative detection of biomarkers based on chemiluminescent nanoparticles and a lensless optical sensor

Cristina Potrich [1,2,*], Gianluca Palmara [1,3,†], Francesca Frascella [3], Lucio Pancheri [4] and Lorenzo Lunelli [1,2]

1 Fondazione Bruno Kessler, Center for Sensors & Devices, via Sommarive 18, I-38123 Trento, Italy; palmara@uji.es (G.P.); lunelli@fbk.eu (L.L.)
2 Consiglio Nazionale delle Ricerche, Istituto di Biofisica, via alla Cascata 56/C, I-38123 Trento, Italy
3 Department of Applied Science and Technology (DISAT), Politecnico di Torino, Corso Duca degli Abruzzi 24, I-10129 Torino, Italy; francesca.frascella@polito.it
4 Department of Industrial Engineering, University of Trento, via Sommarive 9, I-38123 Trento, Italy; lucio.pancheri@unitn.it
* Correspondence: cpotrich@fbk.eu
† Current address: Institute of Advanced Materials (INAM), Universitat Jaume I, Av. Vicente Sos Baynat s/n, 12071 Castellon de la Plana, Spain.

Chemiluminescence reaction

The chemiluminescence signal exploited in this work is generated through the reaction shown in Figure S1A (adapted from the Westar product manual by Cyanagen, www.cyanagen.com). Briefly, the substrate luminol (or one of its derivatives) is converted by the enzyme horseradish peroxidase (HRP) in an intermediate reactive molecule with the help of an enhancer molecule. This intermediate is immediately converted in an excited state product that emits light in the blue region when decays to its ground state.
All the three kits used in this work are based on two-component chemiluminescence substrates: i) the solution A containing the luminol derivative/enhancer solution and ii) the solution B containing the peroxide solution. The specific composition of kit components is patented and different among the three kits.
Luminescence spectra of the three substrates were acquired before (dotted lines, Figure S1B) and after (full lines, Figure S1B) the addition of the HRP enzyme at a final concentration of 16.5 ng/mL. As clearly visible, no signal is present without the enzyme (see the inset of Figure S1B for an enlargement of the y-scale), and the HyperNova kit is the most performant.

A

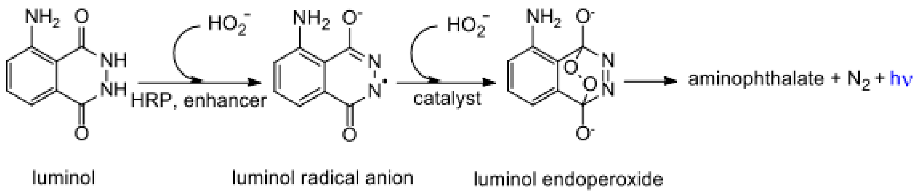

B

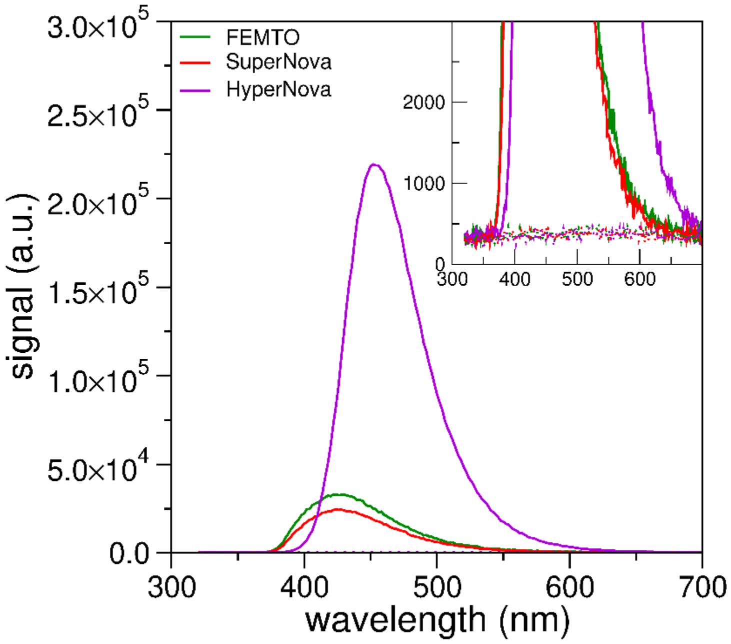

Figure S1. (A) Chemiluminescence catalytic reaction and (B) luminescence spectra of the three developer solutions employed in this work. Dotted lines, before addition of the HRP enzyme. Full lines: after addition of the HRP enzyme.

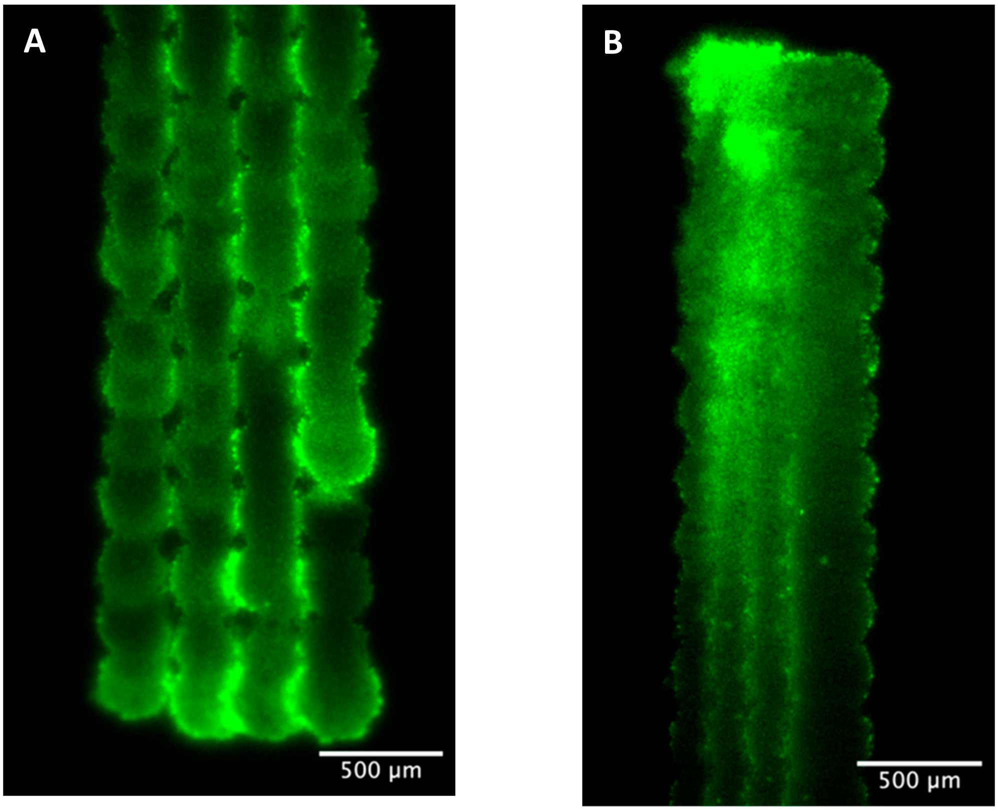

Figure S2. Adjustment of spotting parameters. Fluorescent antibody IgG FITC spotted with Biorad solid pin 310S at 1 mg/mL concentration on nitrocellulose membrane. Spotting parameters: 4x16 spots, ΔY=250 µm. ΔX: 250 µm (panel A), 150 µm (panel B). Images were taken with a Leica DMLA upright fluorescence microscope, 2.5x objective, DFC420C cooled camera.

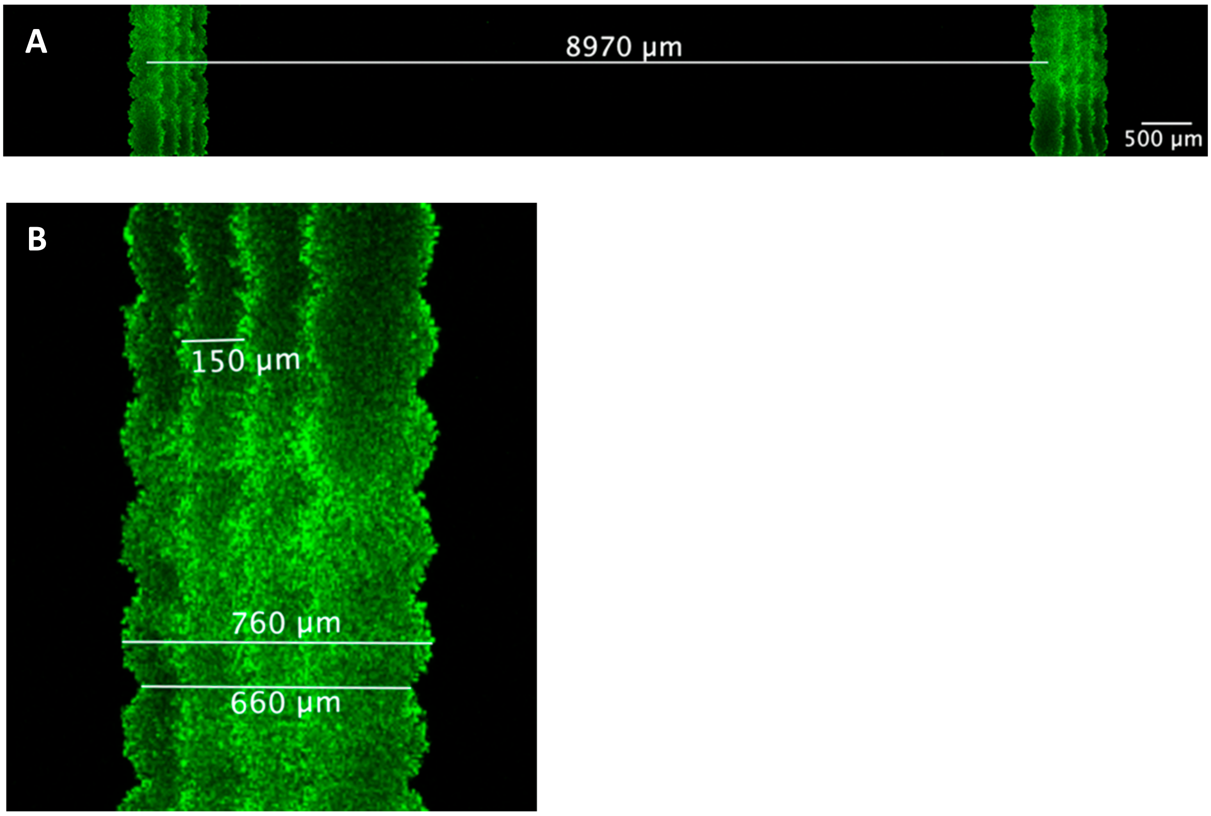

Figure S3. Characterization of spotted lines. Fluorescent antibody IgG FITC spotted at 1 mg/mL concentration on nitrocellulose membrane with two Biorad 310S solid pins. Spotting parameters: 4x16 spots, ΔY=250 µm, ΔX: 150 µm. Images were taken with Leica SP5 confocal microscope with a 10X objective. Panel A shows an image of the whole nitrocellulose strip, obtained by merging images taken by moving the DM6000 stage, using the instrument provided software. Panel B shows a magnification of a line reported in A. Dimensions are superimposed to the pictures.

Atomic force microscopy (AFM) images of gold nanoparticles (gNPs) interacting with conjugate pad (Figure S4) and the nitrocellulose membrane (Figure S5). Data taken in air with a Cypher AFM (Asylum Research, CA, USA) in AC mode, using Olympus AC240TS levers, with a nominal force constant of 2 N/m.

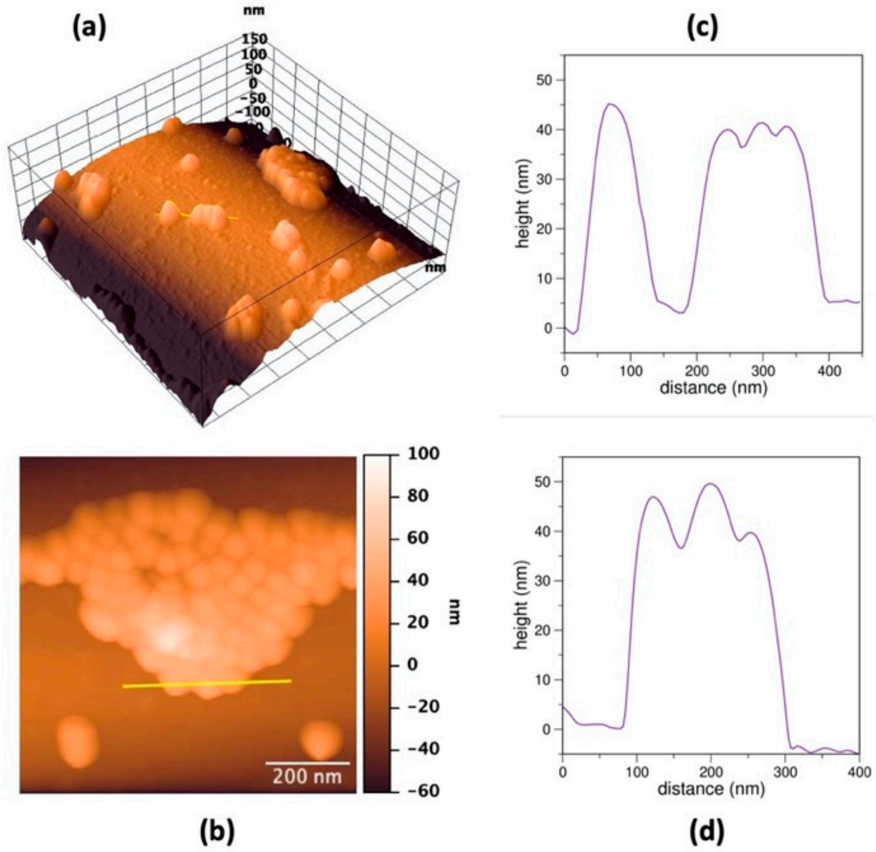

Figure S4. AFM data of the gNPs on the conjugate pad. AFM images of gNPs on a conjugate pad fiber (a and b) and AFM profiles traced along the yellow lines of the same images (c and d).

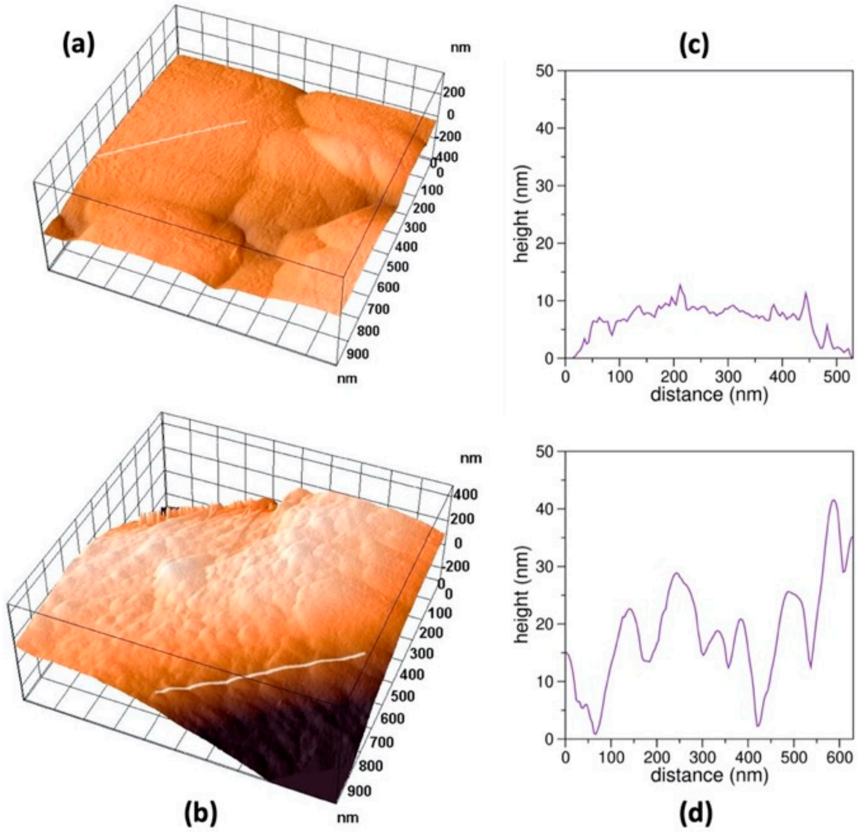

Figure S5. Characterization of the nitrocellulose membrane, previously spotted with protein G and passivated. AFM images of the NC without gNPs (a), and after flowing the gNPs (b). Height profiles traced along the white lines of the same images are reported in panels (c) and (d), respectively.

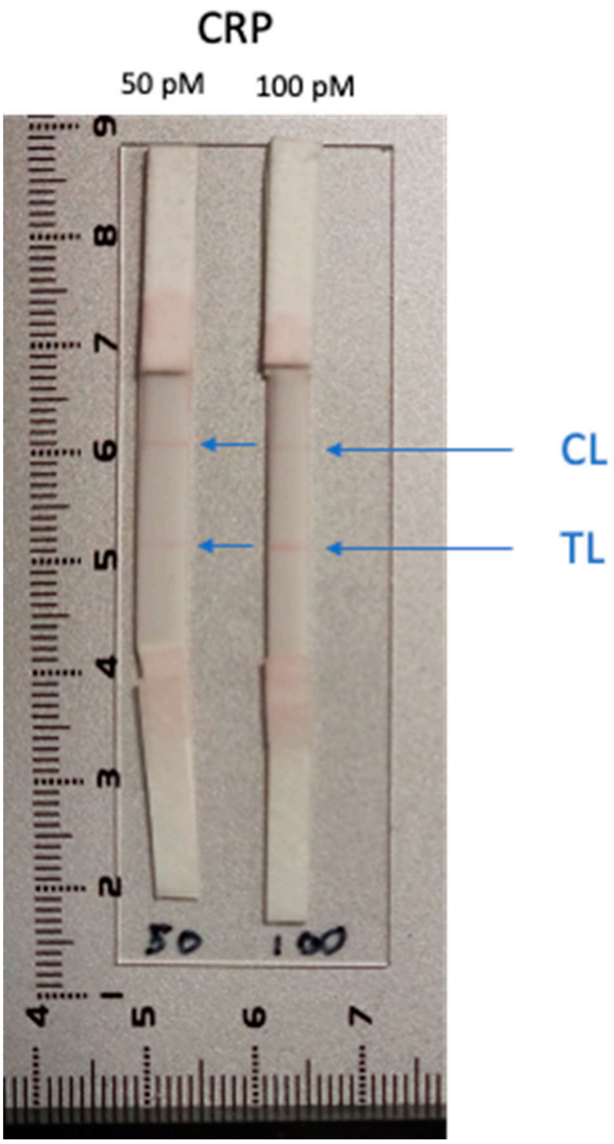

Figure S6. Picture of two LFT runs with high concentration of CRP (left 50 pM, right 100 pM), showing control line (CL) and test line (TL) visible by eye. Blue arrows indicate the lines.

Description	Limit of detection (ng/mL)	Time to results (minutes)	Interferent protein(s)	Reference
CRP Human Instant ELISA™ Kit, Absorbance reader	3×10^{-3} (analytical sensitivity)	130	Plasma, Serum	[40]
Human CRP ELISA Kit (C-Reactive protein), Fluorescence reader	1.95×10^{-3} (sensitivity)	90	Plasma, Serum	[41]
Capillary Elisa	0.1	> 60	Casein (passivation)	[37]
Standard LFT	0.65	-	BSA, IgG (control line)	[38]
Electrochemical detection with two kinds of functional nanoparticles	3.3×10^{-7}	> 120	AFP, CEA, L-Cys, Lysine and UA	[39]
Chemiluminescence detection with SiPM detectors	9.4×10^{-2}	35	BSA (passivation), ProteinG (control line)	This work

Table S1. Comparison of the performances of some CRP detection systems.
